# Changes in volatile compounds of fermented minced pepper during natural and inoculated fermentation process based on headspace–gas chromatography–ion mobility spectrometry

**DOI:** 10.1002/fsn3.1616

**Published:** 2020-05-12

**Authors:** Yuyu Chen, Haishan Xu, Shenghua Ding, Hui Zhou, Dan Qin, Fangming Deng, Rongrong Wang

**Affiliations:** ^1^ College of Food Science and Technology Hunan Agricultural University Changsha China; ^2^ Hunan Provincial Research Center of Engineering and Technology for Fermented Food Changsha China; ^3^ Hunan Agricultural Product Processing Institute Hunan Academy of Agricultural Sciences Changsha China

**Keywords:** fermentation process, fermented minced pepper, headspace–gas chromatography–ion mobility spectrometry, volatile compounds

## Abstract

Changes in volatile compounds of fermented minced pepper (FMP) during natural fermentation (NF) and inoculated fermentation (IF) process were analyzed by the headspace–gas chromatography–ion mobility spectrometry (HS‐GC‐IMS). A total of 53 volatile compounds were identified, including 12 esters, 17 aldehydes, 13 alcohols, four ketones, three furans, two acids, one pyrazine, and one ether. Generally, fermentation time played an important role in volatile compounds of FMP. It was found that most esters, aldehydes, and alcohols obviously decreased with the increase in fermentation time, including isoamyl hexanoate, methyl octanoate, gamma‐butyrolactone, phenylacetaldehyde, methional, and E‐2‐hexenol. Only a few volatile compounds increased, especially for 2‐methylbutanoic acid, 2‐methylpropionic acid, linalool, ethanol, and ethyl acetate. However, no significant difference in volatile compounds was found between NF and IF samples at the same fermentation time. In addition, the fermentation process in all samples was well discriminated as three stages (0 days; 6 day; and 12, 18, and 24 days), and all volatile compounds were divided into two categories (increase and decrease) based on principal component analysis and heat map.

## Graphical abstract

Changes in volatile compounds of FMP during NF and IF process were analyzed by HS‐GC‐IMS. Fermentation time played an important role in volatile compounds of FMP. Most esters, aldehydes, and alcohols obviously decreased, and only a few volatile compounds increased during NF and IF, especially for acids. NF and IF posed little effect on volatile compounds of FMP.

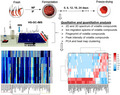

## 1 INTRODUCTION

Pepper (*Capsicum annuum* L.), belonging to the Solanaceae family, is widely planted worldwide and covers approximately 1.99 million ha of harvested area and an annual production of 36.77 million tons in 2018 according to FAO (2018). Pepper fruits are rich in nutrients and bioactive compounds, including *L*‐ascorbic acid, phenolic compounds, carotenoids, and capsaicin, which exhibit antioxidant activities and anti‐inflammatory effects (Hernández‐Carrión et al., 2015; Ribes‐Moya, Raigón, Moreno‐Peris, Fita, & Rodríguez‐Burruezo, 2018). Nowadays, pepper fruits are an important ingredient in several fermented food, including kimchi, fermented pepper paste, and fermented minced pepper (FMP; Li et al., 2019; Wang, Wang, Xiao, Liu, Deng, et al., 2019; Wang, Wang, Xiao, Liu, Jiang, et al., 2019). FMP, as a traditional and local fermented vegetable in the southern regions of China, is widely consumed due to its nutritional and sensory properties (Li, Zhao, et al., 2016). It can be eaten directly or used as cooking ingredient. FMP can be prepared by natural fermentation (NF) and inoculated fermentation (IF). NF was a kind of fermentation methods in which microorganisms from natural environment were used for fermentation, and IF was a kind of fermentation methods in which inoculated microorganisms were used for fermentation. Whatever NF or IF, lactic acid bacteria (LAB) become the dominant microorganism when conditions are suitable for their growth (Sanlier, Gökcen, & Sezgin, 2017). Meanwhile, LAB fermentation involves the production of various metabolites in FMP such as alcohols, organic acids, and active metabolites that contribute to its nutrition, taste, flavor, and functionality (Wang, Wang, Xiao, Liu, Deng, et al., 2019; Wang, Wang, Xiao, Liu, Jiang, et al., 2019).

Flavor plays a key important role in defining sensory and consumer acceptance of FMP. The flavor of FMP is a complex trait, including hot taste from peppers, umami taste from amino acids, and salty taste from NaCl. Previous researchers have focused on flavor characteristics of fermented vegetables products, including fermented peppers, sauerkraut, and kimchi (Sanlier et al., 2017). Wang, Wang, Xiao, Liu, Deng, et al. (2019) showed that alcohols, esters, and ketones were the dominant volatile fractions in fermented/chopped pepper by solid‐phase microextraction and gas chromatography–mass spectrometry, and the fermentation stage was mainly affected by esters, alcohols, aldehydes, and terpenes. Liu et al. (2019) found that the flavor profiles of Sichuan pickle fermented in glass jars (GL), porcelain jars (PO), and plastic jars (PL) were different. The compound with the highest concentration in both PO and GL was the alkanes, while the highest concentration of compound was ester in the PL. Wu et al. (2015) measured changes of flavor compounds in suan cai during NF, and found that there were 17 varieties of volatile flavor components in the early fermentation time, but increased to 57 in the middle fermentation time. In addition, the result also showed that esters and aldehydes were in the greatest diversity and abundance, contributed most to the aroma of suan cai. Kang and Baek (2014) found that 19 aroma‐active compounds were detected by aroma extract dilution analysis in Korean fermented red pepper paste (*gochujang*), and 12 aroma‐active compounds were detected by headspace–solid‐phase microextraction–gas chromatography–olfactometry. Hence, the variety and content of volatile compounds in fermented vegetables could be affected by multiple factors, such as raw material, container, and fermentation time. For FMP, the previous research and industrial production mainly focused on the optimization of process parameters, including raw material, NaCl and CaCl_2_ content, LAB inoculum, fermentation temperature, and time based on the change of quality. However, knowledge about changes in flavor compounds of FMP at different stages with NF or IF is not available.

Ion mobility spectrometry (IMS), a rapid detection technique, was used to detect the gasified volatile compounds by ion separation based on their ion mobility velocity (Zhang et al., 2016). The detection technology presented many advantages, including easy operation, high analysis speed, high sensitivity, and no complex sample preparation steps. However, its analysis characteristics were often limited for complex samples, especially for complex systems in food and agricultural products (Arce et al., 2014). Combining IMS with other instruments is a more suitable and effective way to make better use of its advantages. Recently, headspace–gas chromatography–ion mobility spectrometry (HS‐GC‐IMS) has been applied in detecting volatile compounds of fruits and vegetables, such as candied kumquats (Hu et al., 2019), jujube fruits (Yang et al., 2019), and dried peppers (Ge et al., 2020). As a consequence, HS‐GC‐IMS is effective to identify the flavor characteristic of fruits and vegetables. However, the changes in characteristic compounds linked to the flavors of FMP during NF and IF process are still not available. Hence, HS‐GC‐IMS can be used to establish fingerprints of volatile compounds in FMP during NF and IF process.

In this study, the changes of volatile compounds in FMP during NF and IF process were analyzed, and several target volatile compounds in samples were detected using HS‐GC‐IMS, principal component analysis (PCA), and the heat map. The results confirmed the potential of HS‐GC‐IMS to identify the volatile compound characteristics and provided a rapid method to determine the flavor quality of FMP during fermentation process.

## 2 MATERIALS AND METHODS

### 2.1 Materials

Fresh peppers (*Capsicum annuum* L.) cv. “yanhong” were grown in an experimental field of Gaoqiao Town, Changsha County, Hunan Province, China (N28°28′38.08, E113°20′54.65″). All harvested peppers were up to commercial maturity. After harvesting, the peppers were delivered to the laboratory immediately. Peppers with uniformity of size, color, and weight, free from visible blemishes, disease, and/or physical damage, were selected as the experiment raw materials. W‐4 LAB was isolated, purified, and extended culture from our previous FMP obtained by NF, and it posed strong resistance to high salt and acid.

### 2.2 Preparation of FMP

Selected peppers were removed handle, washed, drained, and minced into small pieces (0.5–1 cm × 0.5–1 cm), and 10% (w/w) NaCl and 0.1% CaCl_2_ (w/w) were added to the minced peppers and then stirred for 5 min. Above samples were divided into two groups: One group inoculated with 5% (w/w) W‐4 LAB (10^7^ CFU/ml) was regarded as IF, and another group without inoculated LAB was regarded as NF. Prepared samples were put into pickle jars, and the jars were sealed with water to exclude air and fermented in a 30°C incubator. FMPs were obtained on 0, 6, 12, 18, and 24 days, respectively. The collected samples were detected immediately after freeze‐drying and grinding.

### 2.3 HS‐GC‐IMS analysis

All the analyses were obtained by HS‐GC‐IMS instrument and related supplementary analysis software according to the method of Sun et al. (2019) with some modifications. Freeze‐dried (0.5 g) FMP was weighted and then transferred into a 20‐ml headspace bottle. The FMP was incubated at 80°C in the headspace bottle, with the speed of 500 rpm for 10 min. After incubation, 500 μl headspace was automatically injected using a heated syringe at 85°C into a FS‐SE‐54‐CB‐1 (15 m × 0.53 mm ID) capillary column. The carried gas during injection was nitrogen (99.99% purity), which was under the below programmed flow to carry samples: 2 ml/min held for 2 min, flowed ramp from 2 ml/min to 100 ml/min in 18 min, and then maintained 100 ml/min for 10 min until stopping. The analyses were separated in the column at 60°C and then ionized in the IMS ionization chamber at 45°C. The constant flow of drift gas flow was set up to 150 ml/min. The instrument was standardized by linear retention index (RI) of n‐ketones, for the reason that IMS had no response to alkanes. Calculation of RI of volatile compounds was based on the n‐ketones C_4_‐C_9_. Comparing the drift time and RI in the GC‐IMS library, volatile compounds in NF and IF samples were well identified. The qualitative analysis of volatile compounds was conducted based on the IMS and NIST database built in GC × IMS Library Search. The quantitative analysis for volatile compounds was mainly based on the peak intensity in HS‐GC‐IMS, and the peak intensity was proportional to the content of volatile compounds.

### 2.4 Data analysis

All the experiment was performed in triplicate. The spectra were analyzed with laboratory analytical viewer (LAV), and the difference profiles and fingerprints of volatile compounds were constructed with the Reporter and Gallery plug‐ins. The NIST and IMS database were built into the software for qualitative analysis of samples. The line and bar charts drawn by Origin 2018 were used to analyze change of volatile compounds in FMP during fermentation process. PCA obtained by the original date was used for clustering analysis of principal compounds in samples. The heat map was generated using the heat map plug‐in of origin 2018, and the methods used for clustering of original date were Ward minimum variance and Euclidean distance.

## 3 RESULTS AND DISCUSSION

### 3.1 Changes of HS‐GC‐IMS spectra in FMP

**FIGURE 1:**
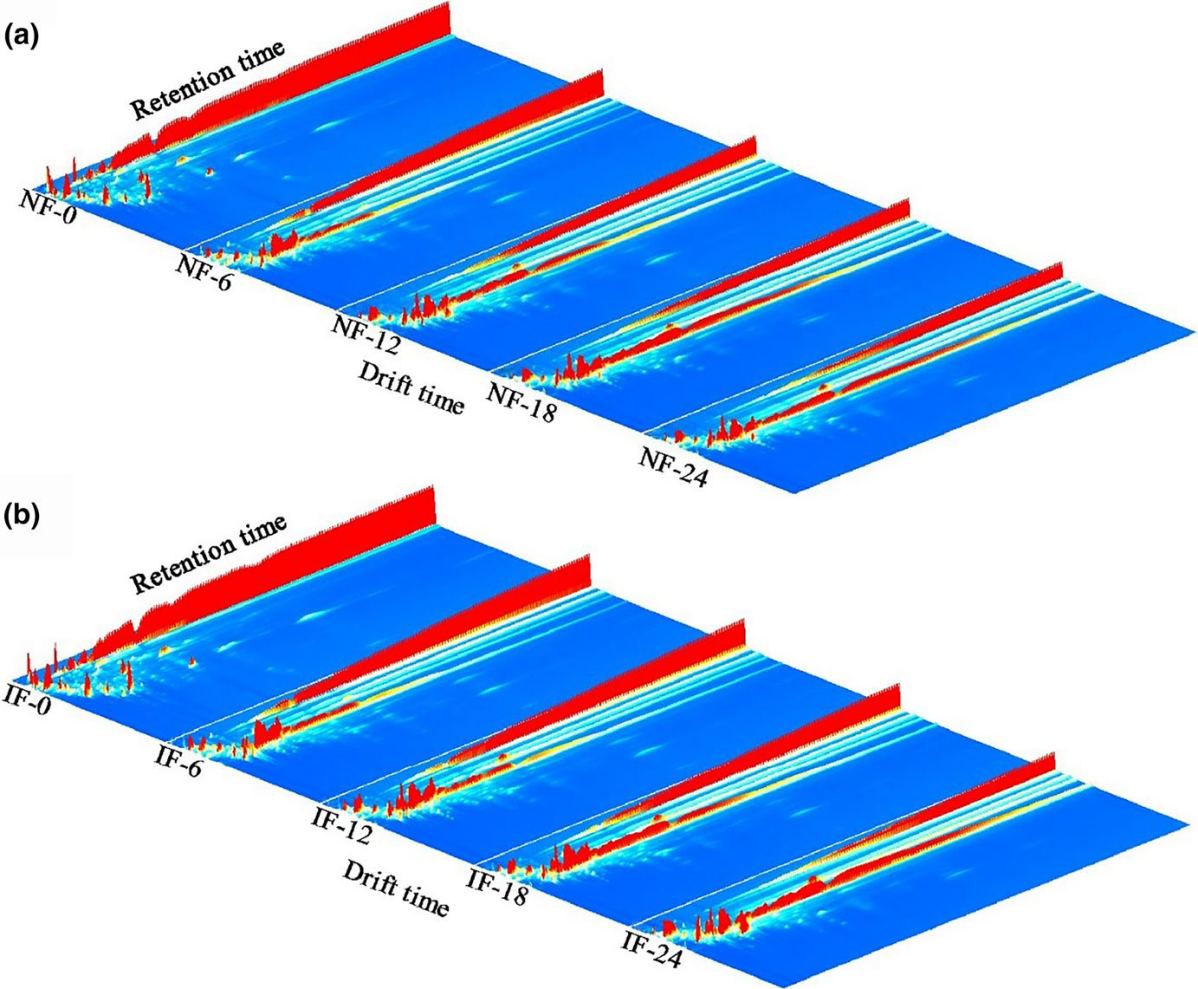
Changes in 3D topography of volatile compounds during NF and IF process. (a) NF; (b) IF. IF: inoculated fermentation; NF: natural fermentation

The volatile compounds of FMP during NF and IF were detected by HS‐GC‐IMS. The data were exhibited with the 3D spectrum, where the *x*‐axis represented the ion migration time, the *y*‐axis represented the retention time of the gas chromatograph, and the *z*‐axis represented the peak intensity. As shown in Figure 1, there was the similar peak signal distribution in all samples during fermentation process. This phenomenon suggested that all FMP posed the same volatile compounds during fermentation process. However, the peak signal intensity showed some differences in FMP during fermentation process. It indicated that content of volatile compounds changed (increasing or decreasing) with the prolonging of fermentation time. Yu et al. (2013) proved that the prominent microorganisms posed clear relationships with changes in flavor during fermentation. However, no obvious differences in volatile compounds were found between NF and IF samples. It was found that the signal intensities of almost all volatile compounds in IF samples were similar to those in NF ones. This phenomenon showed that two fermentation methods played minor effects on changes in volatile compounds during fermentation process.

**FIGURE 2:**
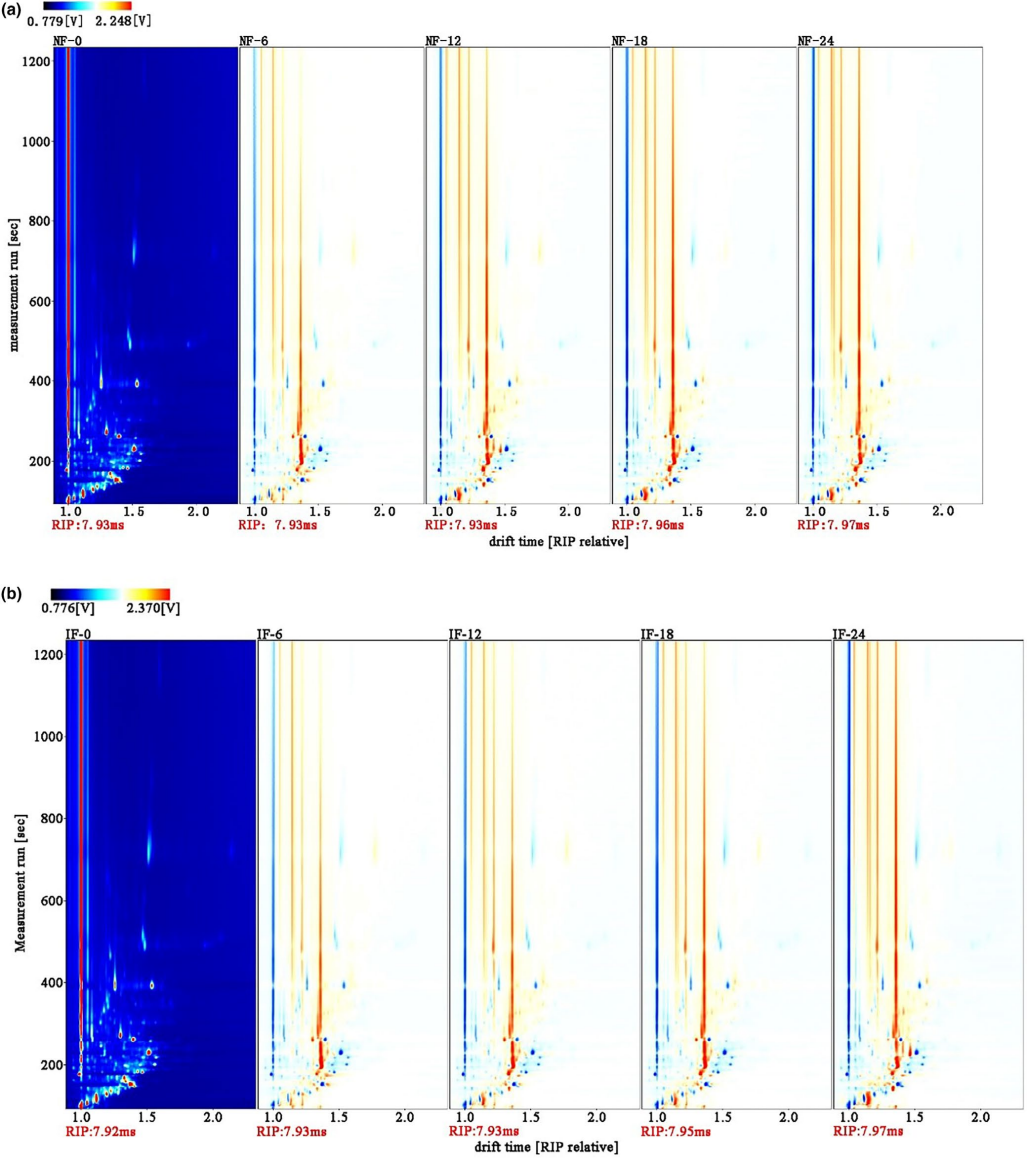
Changes in 2D topography of volatile compounds during NF and IF process. (a) NF; (b) IF. IF: inoculated fermentation; NF: natural fermentation

The volatile compounds in FMP were not easy for analysis by 3D spectra. Hence, the 2D spectra were used for further comparison, as shown in Figure 2. The reactive ion peak (RIP) was exhibited with the red vertical line at the horizontal coordinate of 1.0. The normalized migration time, from 7.92 to 7.97 ms, was used to avoid the change of ion migration time caused by temperature and pressure deviation during detection process. For comparing with the differences in volatile compounds in all samples, fresh samples were taken as the reference, and the spectral background colors of other samples were white after deducting that of original samples. In 2D spectra, every dot on the right side of RIP represented the specific volatile compound. The color of dots represented the concentration of volatile compounds. The blue dots represented that the volatile compounds posed lower concentration comparing with those of the control, and the red dots represented that the volatile compounds posed higher concentration comparing with those of the control. As shown in Figure 2a,b, most dots were located in 2D spectra area from 0 to 400 s of retention time and from 1.0 to 1.5 of drift time, and few dots were located in 2D spectra area from 400 to 1,200 s of retention time.

As shown in Figure 2a,b, the total number of dots in 2D spectra hardly changed. However, the number of blue or red dots changed (increase or decrease). These results indicated that the varieties of volatile compounds in samples were the same, but the content of volatile compounds changed with the prolonging of fermentation time. The reason was that metabolism and decomposition of microorganisms caused volatile compounds to increase or decrease during fermentation process. Wu et al. (2015) showed that various bacteria and fungi, especially for LAB and yeast, possessed obvious correlation with the changes in volatile compounds during fermentation process. In addition, some pathways of biosynthesis and degradation also existed in FMP, including EMP pathway, Strecker pathway, and decarboxylation pathway, which could increase volatile compound content (Kang & Baek, 2014; Li, Zhao, et al., 2016; Li, Dong, Huang, & Wang, 2016). However, the color of red or blue dots in NF and IF samples was similar to each other. Therefore, the content of volatile compounds in NF and IF samples was almost the same. This result was because of the fact that LAB was the prominent microorganism under suitable conditions whatever NF or IF.

### 3.2 Qualitative analysis of volatile compounds in FMP

**FIGURE 3:**
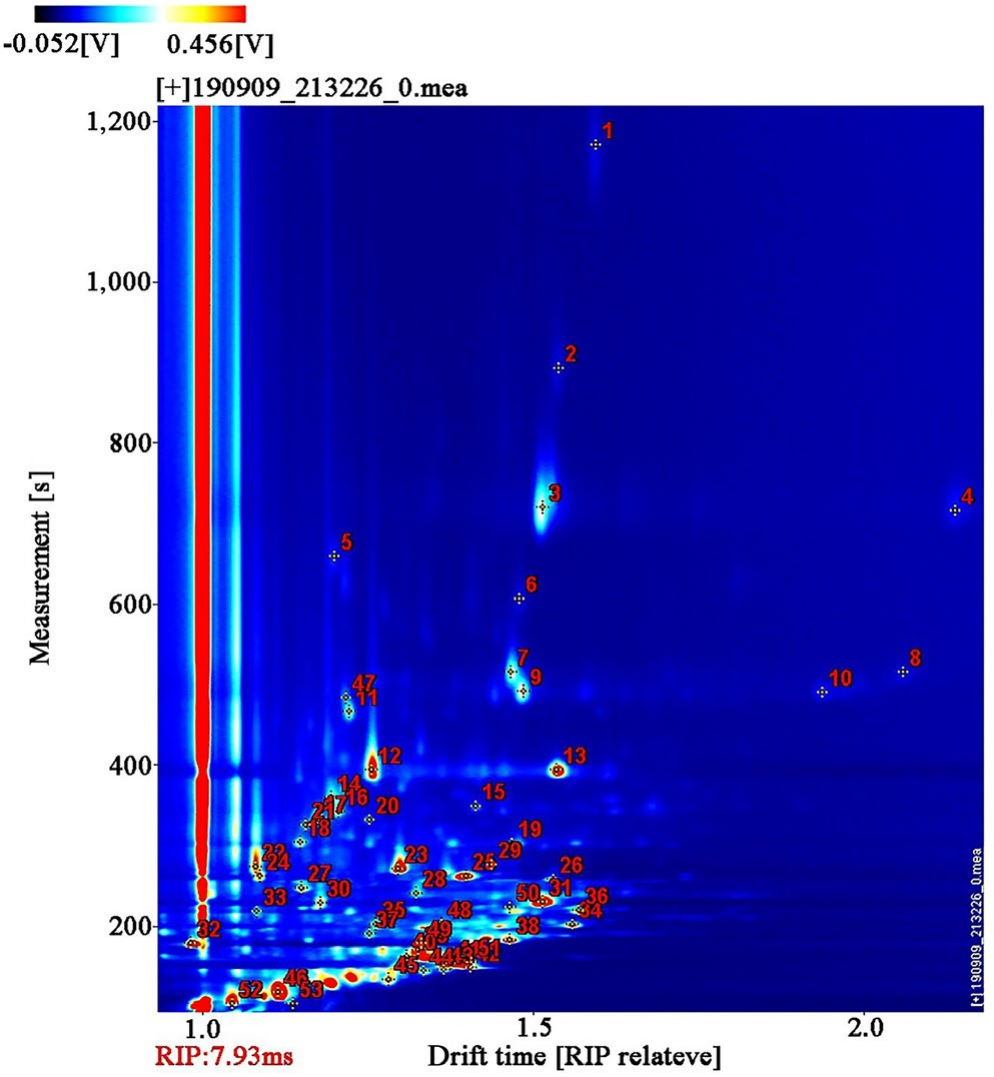
Ion migration spectra of volatile compounds identified by HS‐GC‐IMS during NF and IF process. IF: inoculated fermentation; NF: natural fermentation

The qualitative analysis of volatile compounds in FMP during fermentation process was represented by numbers, as shown in Figure 3 and Table 1. Each dot represented a type of volatile compound. The marked dots were identified volatile compounds, but unmarked dots were nonidentified volatile compounds. All FMP showed 53 identified dots by GC × IMS library analysis. Therefore, there were the same volatile compounds in all samples. This result indicated that fermentation methods hardly affected varieties of volatile compounds during fermentation process. In Figure 3 and Table 1, a total of 43 typical volatile compounds, including nine esters, 12 aldehydes, 11 alcohols, four ketones, three furans, two acids, one pyrazine, and one ether, were identified by NIST and IMS database in all samples. However, monomer and dimer of the same volatile compound exhibited similar retention time, but different migration time. The volatile compounds with high content or high proton affinity were beneficial for the production of new dimer (Arroyo‐Manzanaresa et al., 2018; Lantsuzskaya, Krisilov, & Levina, 2015). Arroyo‐Manzanaresa et al. (2018) found that several monomers with proton affinity absorbed some reactant protons, and then formed dimer by the combination of different monomers. Meanwhile, several volatile compounds could produce different signals, consisting of isoamyl hexanoate, methyl octanoate, nonanal, phenylacetaldehyde, heptadienal, benzaldehyde, gamma‐butyrolactone, methional, E‐2‐hexenol, hexanal, methylbutanal, methylbutanol, and ethanol. Li et al. (2010) showed that the same volatile compounds with different concentrations also might produce multiple signals in *tricholoma matsutake*. In addition, new formed dimer showed higher molecular weight than the monomer, and further generated multiple signals.

**TABLE 1:** Information of identified volatile compounds during NF and IF process

Count	Compound	CAS#	Formula	MW	RI	Rt (s)	Dt (RIPrel)	Comment	Identification
1	Hexyl hexanoate	C6378650	C_12_H_24_O_2_	200.3	1,558.7	1,170.184	1.5963		RI,Dt
2	Ethyl nonanoate	C123295	C_11_H_22_O_2_	186.3	1,374.8	892.49	1.5408		RI,Dt
3	Isoamyl hexanoate	C2198610	C_11_H_22_O_2_	186.3	1,260.8	720.352	1.5153	Monomer	RI,Dt
4	Isoamyl hexanoate	C2198610	C_11_H_22_O_2_	186.3	1,257.5	715.481	2.1399	Dimer	RI,Dt
5	Methyl salicylate	C119368	C_8_H_8_O_3_	152.1	1,220.3	659.302	1.2006		RI,Dt
6	Ethyl octanoate	C106321	C_10_H_20_O_2_	172.3	1,185.2	606.274	1.4812		RI,Dt
7	Methyl octanoate	C111115	C_9_H_18_O_2_	158.2	1,124.9	515.216	1.468	Monomer	RI,Dt
8	Methyl octanoate	C111115	C_9_H_18_O_2_	158.2	1,124.5	514.614	2.0618	Dimer	RI,Dt
9	Nonanal	C124196	C_9_H_18_O	142.2	1,108.9	491.123	1.4867	Monomer	RI,Dt
10	Nonanal	C124196	C_9_H_18_O	142.2	1,108.5	490.521	1.9397	Dimer	RI,Dt
11	Methyl benzoate	C93583	C_8_H_8_O_2_	136.1	1,092.5	466.428	1.2237		RI,Dt
12	Phenylacetaldehyde	C122781	C_8_H_8_O	120.2	1,043.3	394.148	1.2576	Monomer	RI,Dt
13	Phenylacetaldehyde	C122781	C_8_H_8_O	120.2	1,042.9	393.546	1.5376	Dimer	RI,Dt
14	E‐E‐2‐4‐Heptadienal	C4313035	C_7_H_10_O	110.2	1,016.8	358.741	1.196		RI,Dt
15	Octanal	C124130	C_8_H_16_O	128.2	1,008.3	348.385	1.4139		RI,Dt
16	E‐Z‐2‐4‐Heptadienal	C4313024	C_7_H_10_O	110.2	1,002.6	341.766	1.2064		RI,Dt
17	6‐Methyl‐5‐hepten‐2‐one	C110930	C_8_H_14_O	126.2	995.3	333.382	1.1743		RI,Dt
18	Benzaldehyde	C100527	C_7_H_6_O	106.1	965.2	303.377	1.1489	Monomer	RI,Dt
19	Benzaldehyde	C100527	C_7_H_6_O	106.1	964.7	302.936	1.4701	Dimer	RI,Dt
20	2‐Pentylfuran	C3777693	C_9_H_14_O	138.2	994.0	332.058	1.2533		RI,Dt
21	1‐Octen‐3‐ol	C3391864	C_8_H_16_O	128.2	987.5	324.998	1.1569		RI,Dt
22	Gamma‐butyrolactone	C96480	C_4_H_6_O_2_	86.1	927.0	272.937	1.0809	Monomer	RI,Dt
23	Gamma‐butyrolactone	C96480	C_4_H_6_O_2_	86.1	924.0	270.819	1.2971	Dimer	RI,Dt
24	Methional	C3268493	C_4_H_8_OS	104.2	909.5	261.135	1.0882	Monomer	RI,Dt
25	Methional	C3268493	C_4_H_8_OS	104.2	910.5	261.74	1.4003	Dimer	RI,Dt
26	2‐6‐Dimethylpyrazine	C108509	C_6_H_8_N_2_	108.1	904.4	257.806	1.5315		RI,Dt
27	Cyclohexanone	C108941	C_6_H_10_O	98.1	887.1	247.358	1.1514		RI,Dt
28	1‐Hexanol	C111273	C_6_H_14_O	102.2	876.0	241.002	1.3241		RI,Dt
29	2‐Acetylfuran	C1192627	C_6_H_6_O_2_	110.1	932.1	276.569	1.4392		RI,Dt
30	E‐2‐Hexenol	C928950	C_6_H_12_O	100.2	852.7	228.451	1.1793	Monomer	RI,Dt
31	E‐2‐Hexenol	C928950	C_6_H_12_O	100.2	855.0	229.661	1.5157	Dimer	RI,Dt
32	Dimethyl disulfide	C624920	C_2_H_6_S_2_	94.2	743.1	178.214	0.9837		RI,Dt
33	Furfurol	C98011	C_5_H_4_O_2_	96.1	831.3	217.693	1.0831		RI,Dt
34	Hexanal	C66251	C_6_H_12_O	100.2	796.8	201.459	1.5618	Dimer	RI,Dt
35	Hexanal	C66251	C_6_H_12_O	100.2	797.2	201.665	1.2638	Monomer	RI,Dt
36	3‐Methylpentanol	C589355	C_6_H_14_O	102.2	833.4	218.721	1.5706		RI,Dt
37	1‐Pentanol	C71410	C_5_H_12_O	88.1	770.0	189.54	1.2531		RI,Dt
38	2‐Methylbutanol	C137326	C_5_H_12_O	88.1	754.2	182.759	1.4661		RI,Dt
39	Acetoin	C513860	C_4_H_8_O_2_	88.1	722.3	170.019	1.3273		RI,Dt
40	2‐Ethylfuran	C3208160	C_6_H_8_O	96.1	699.3	161.799	1.3107		RI,Dt
41	1‐Butanol	C71363	C_4_H_10_O	74.1	671.5	153.168	1.3781		RI,Dt
42	3‐Methylbutanal	C590863	C_5_H_10_O	86.1	653.7	148.27	1.4073		RI,Dt
43	2‐Methylpropanol	C78831	C_4_H_10_O	74.1	642.2	145.31	1.3662		RI,Dt
44	Ethyl acetate	C141786	C_4_H_8_O_2_	88.1	637.6	144.153	1.3364		RI,Dt
45	Butanal	C123728	C_4_H_8_O	72.1	594.7	133.973	1.283		RI,Dt
46	Acetone	C67641	C_3_H_6_O	58.1	524.9	117.893	1.1158		RI,Dt
47	Linalool	C78706	C_10_H_18_O	154.3	1,103.6	483.186	1.218		RI,Dt
48	2‐Methylpropionic acid	C79312	C_4_H_8_O_2_	88.1	798.3	202.172	1.363		RI,Dt
49	3‐Methylbutanol	C123513	C_5_H_12_O	88.1	744.5	178.74	1.3325		RI,Dt
50	2‐Methylbutanoic acid	C116530	C_5_H_10_O_2_	102.1	843.4	223.707	1.4662		RI,Dt
51	2‐Methylbutanal	C96173	C_5_H_10_O	86.1	675.7	154.407	1.4087		RI,Dt
52	Ethanol	C64175	C_2_H_6_O	46.1	458.1	102.494	1.0455	Monomer	RI,Dt
53	Ethanol	C64175	C_2_H_6_O	46.1	461.2	103.211	1.1372	Dimer	RI,Dt

Note

The ordinal numbers in Table 1 corresponded to those in Figure 3.

Abbreviations: Dt: drift time; IF: inoculated fermentation; MW: molecular mass; NF: natural fermentation; RI: retention index; Rt: retention time.

### 3.3 Fingerprint analysis of volatile compounds in FMP

**FIGURE 4:**
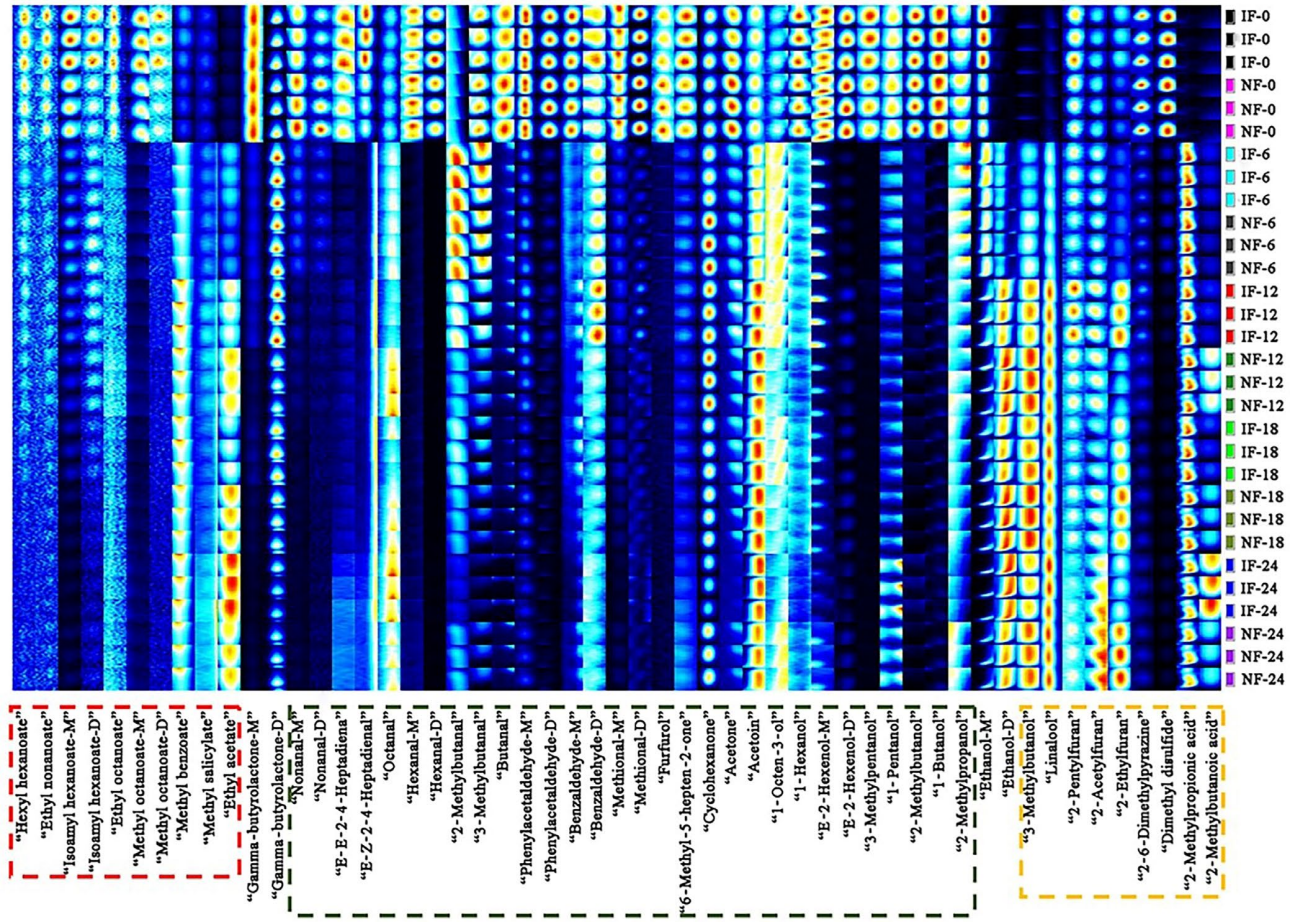
Changes in fingerprint of volatile compounds during NF and IF process. D: dimer; IF: inoculated fermentation; IF‐0, IF‐6, IF‐12, IF‐18, IF‐24: IF samples were obtained on 0, 6, 12, 18, and 24 days; M: monomer; NF: natural fermentation; NF‐0, NF‐6, NF‐12, NF‐18, NF‐24: NF samples were obtained on 0, 6, 12, 18, and 24 days

Although the 3D and 2D spectrum presented the change tendency of volatile compounds during NF and IF, the specific volatile compounds were not accurately judged. Hence, all signal peaks were used to make a detailed fingerprint analysis, as shown in Figure 4. In fingerprints, each column represented a kind of volatile compounds, each row represented the FMP samples, and volatile compound content was determined by the brightness degree of color.

In the raw materials (fermented on 0 days), high content of esters, aldehydes, and alcohols were found, especially for isoamyl hexanoate, gamma‐butyrolactone, phenylacetaldehyde, methional, E‐2‐hexanol, and 1‐butanol. The change in volatile compounds during NF and IF was presented in different color frames. In the red frame, the content of volatile compounds, such as hexyl hexanoate, ethyl nonanoate, isoamyl hexanoate, ethyl octanoate, and methyl octanoate, decreased to a low level. The decrease in above volatile compounds was related to the accumulation of acids. High acid condition induced by microorganisms could accelerate the hydrolysis of some volatile compounds (Bautista‐Expósito, Peñas, Silván, Frias, & Martínez‐Villaluenga, 2018; Tomita, Nakamura, & Okada, 2018). In addition, the content of several volatile compounds increased to a high level, such as ethyl acetate. However, the fingerprint brightness of above volatile compounds in NF samples was similar to those in IF samples during fermentation process. In the green frame, many volatile compounds sharply decreased with the prolonging of fermentation time, and showed low signal intensity and gray color in the late fermentation time. These volatile compounds were mainly aldehydes (nonanal, E‐Z‐2‐4‐heptadienal, hexanal, methylbutanal, etc.), ketones (acetone, acetoin, etc.), and alcohols (methylpentanol, 1‐butanol, etc.). This result was slightly different from the results of Wang, Wang, Xiao, Liu, Deng, et al. (2019), which might be related to pepper species, fermented methods, and the concentration of salt. Otherwise, the content of above volatile compounds showed similar fingerprint between NF and IF samples. In the yellow frame, the brightness degree of color in alcohols (linalool), furans (2‐ethylfuran, 2‐acetylfuran), and acids (2‐methylpropionic acid, 2‐methylbutanoic acid) increased with the prolonging of fermentation time, which indicated that these volatile compound content increased to a maximal level at the end of NF or IF. However, these volatile compounds in IF samples posed similar content with those in NF samples during fermentation process. Above result was accordance with the 3D and 2D spectra analysis. Moreover, the content of 2‐pentylfuran showed a fluctuate trend. To compare with volatile compound content in NF and IF samples during fermentation process, peak intensity values about volatile compounds needed further exploration.

### 3.4 Changes in volatile compounds of FMP

**FIGURE 5:**
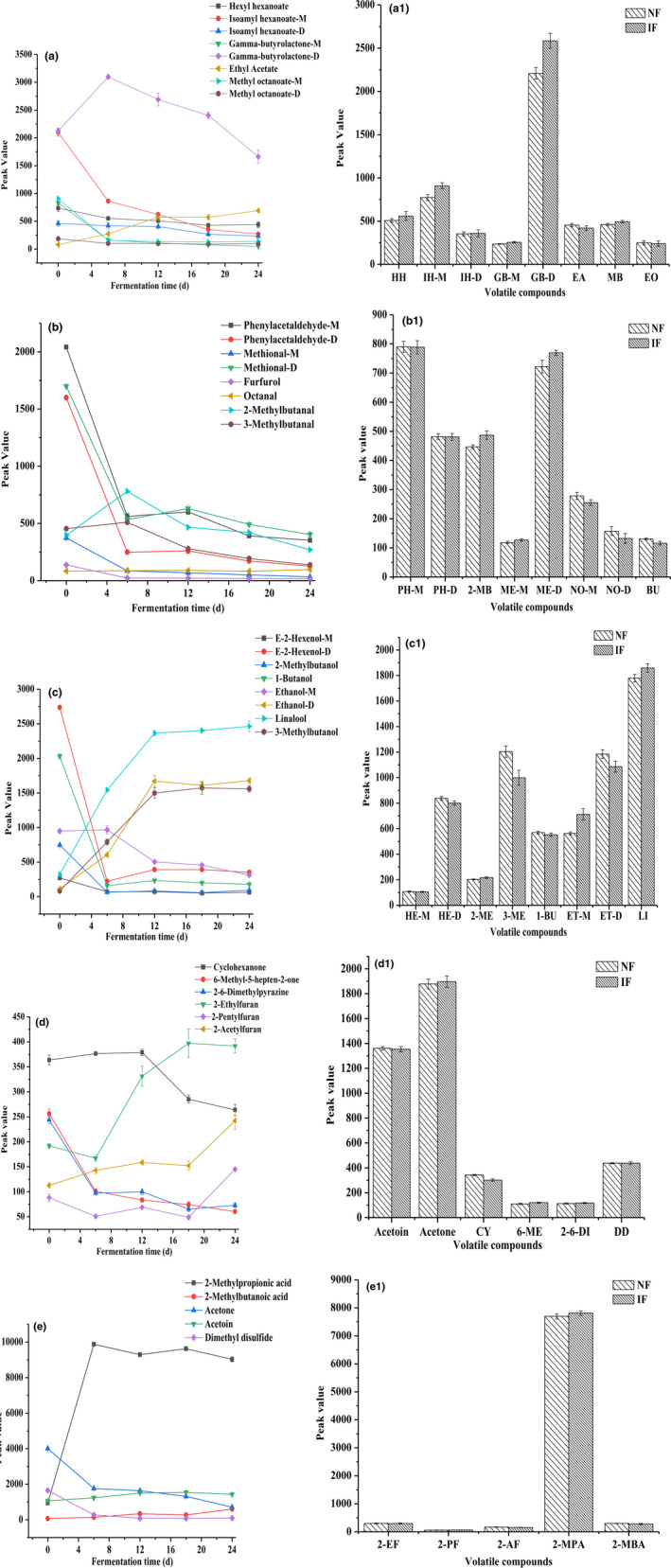
Changes in peak volume of volatile compounds during NF and IF process. NF: natural fermentation; IF: inoculated fermentation; (a, a_1_) esters; (b, b_1_) aldehydes; (c, c_1_) alcohols; (d, d_1_, e, e_1_) other volatile compounds (ketones, furans, acids, pyrazine, and ether); a, b, c, d, and e showed changes in peak volume of volatile compounds during NF and IF process; a_1_, b_1_, c_1_, d_1_, and e_1_ showed peak volume differences in volatile compounds between NF and IF samples. 1‐BU: 1‐butanol; 2‐6‐DI: 2‐6‐dimethylpyrazine; 2‐AF: 2‐acetylfuran; 2‐EF: 2‐ethylfuran; 2‐MBA: 2‐methylbutanoic acid; 2‐ME: 2‐methylbutanal; 2‐ME: 2‐methylbutanol; 2‐MPA: 2‐methylpropionic acid; 2‐PF: 2‐pentylfuran; 3‐ME: 3‐methylbutanol; 6‐ME: 6‐methyl‐5‐hepten‐2‐one; BU: butanal; CY: cyclohexanone; DD: dimethyl disulfide; EA: ethyl acetate; EO: ethyl octanoate; ET‐D: ethanol‐D; ET‐M: ethanol‐M; GB‐D: gamma‐butyrolactone‐D; GB‐M: gamma‐butyrolactone‐M; HE‐D: E‐2‐hexenol‐D; HE‐M: E‐2‐hexenol‐M; HH: hexyl hexanoate; IH‐D: isoamyl hexanoate‐D; IH‐M: isoamyl hexanoate‐M; LI: linalool; MB: methyl benzoate; ME‐D: methional‐D; ME‐M: methional‐M; NO‐D: nonanal‐D; NO‐M: nonanal‐M; PH‐D: phenylacetaldehyde‐D; PH‐M: phenylacetaldehyde‐M

Seven kinds of volatile compounds, including eight esters, eight aldehydes, eight alcohols, and 11 other volatile compounds (four ketones, three furans, two acids, one ether, and one pyrazine), were used to explore the variation during NF and IF. Vegetable fermentation could be divided into aerobic fermentation and anaerobic fermentation (Gobbetti, Di Cagno, & De Angelis, 2010). The production of FMP was mainly anaerobic fermentation, such as alcohol and lactic acid. As shown in Figure 5a–d and Table 2, most of volatile compound content decreased with prolonging of fermentation time, only several volatile compounds increased. The reason for this phenomenon was that the growth of many microorganisms in FMP was inhibited by high concentration of salt, low pH, and oxygen‐deficient environment, so reduced the development of many volatile compounds. However, LAB posed a strong ability to resist adverse environment and produced volatile compounds by fermentation (homo‐ and heterofermentative LAB fermentation), especially for organic acids and alcohols (Esteban‐Torres et al., 2015; Yu et al., 2013). The followed analysis was used to further explore the specific change in various types of volatile compounds.

**TABLE 2:** The peak volume values of volatile compounds during NF and IF process

Compound	NF					IF				
	0 days	6 days	12 days	18 days	24 days	0 days	6 days	12 days	18 days	24 days
Esters										
Hexyl hexanoate	696.30 ± 20.34a	559.14 ± 33.24b	434.00 ± 12.77c	408.59 ± 3.46c	436.06 ± 41.10c	779.21 ± 95.42a	542.20 ± 28.10bc	579.30 ± 51.81b	448.62 ± 14.80c	443.13 ± 69.57c
Ethyl nonanoate	600.66 ± 41.68a	407.66 ± 5.54b	425.48 ± 24.07b	387.19 ± 31.80b	427.15 ± 85.05b	628.67 ± 121.40a	349.95 ± 96.91b	423.90 ± 35.68b	393.83 ± 64.64b	406.79 ± 34.57b
Isoamyl hexanoate‐M	1,891.90 ± 76.32a	906.95 ± 24.72b	502.06 ± 12.55c	277.58 ± 37.58d	289.29 ± 20.52d	2,308.16 ± 63.08a	817.42 ± 38.20b	744.71 ± 31.23c	430.00 ± 12.28d	246.64 ± 27.50e
Isoamyl hexanoate‐D	406.63 ± 21.23b	477.36 ± 42.08a	391.57 ± 30.30b	242.91 ± 7.21c	245.05 ± 12.14c	515.36 ± 69.63a	360.54 ± 77.93bc	414.34 ± 11.48b	291.81 ± 16.11cd	216.06 ± 28.54d
Methyl salicylate	184.20 ± 9.83b	179.81 ± 13.57b	213.58 ± 15.25a	172.11 ± 21.31b	182.27 ± 17.92b	181.85 ± 24.87b	166.66 ± 12.82b	243.66 ± 6.86a	190.73 ± 14.17b	194.66 ± 17.17b
Ethyl octanoate	309.48 ± 14.18a	236.60 ± 18.51b	243.39 ± 29.63b	237.88 ± 19.48b	224.92 ± 32.42b	317.71 ± 48.60a	204.26 ± 50.24b	225.48 ± 23.26b	225.25 ± 6.30b	227.38 ± 23.10b
Methyl octanoate‐M	832.48 ± 8.71a	157.87 ± 6.11b	131.33 ± 12.74cd	117.48 ± 7.20d	136.08 ± 12.27c	977.04 ± 11.39a	159.16 ± 17.56b	133.20 ± 7.43b	138.95 ± 18.19b	138.54 ± 5.71b
Methyl octanoate‐D	166.44 ± 10.03a	108.47 ± 10.16b	94.36 ± 1.57bc	83.73 ± 2.62c	100.26 ± 8.83b	204.37 ± 40.59a	111.87 ± 1.52b	103.89 ± 2.62b	90.41 ± 9.75b	96.38 ± 17.43b
Gamma‐butyrolactone‐M	825.36 ± 2.72a	164.00 ± 2.65b	75.09 ± 6.86c	61.10 ± 3.34d	54.93 ± 2.55d	839.73 ± 18.28a	160.32 ± 5.11b	140.78 ± 11.56b	91.15 ± 9.38c	43.18 ± 1.68d
Gamma‐butyrolactone‐D	2,129.95 ± 31.33b	2,946.32 ± 19.31a	2,071.85 ± 144.52b	2,165.19 ± 82.49b	1,725.34 ± 58.30c	2,117.70 ± 70.64c	3,250.77 ± 62.68a	3,309.24 ± 86.57a	2,646.14 ± 37.57b	1,598.90 ± 174.77d
Methyl benzoate	211.83 ± 7.43d	421.04 ± 17.80c	566.03 ± 23.24a	570.57 ± 9.50a	534.35 ± 9.30b	276.18 ± 12.41c	413.70 ± 5.22b	575.99 ± 14.03a	590.11 ± 33.00a	608.06 ± 8.19a
Ethyl Acetate	81.35 ± 2.18d	238.55 ± 5.48c	656.58 ± 52.67ab	689.45 ± 32.42a	606.97 ± 3.23b	75.79 ± 5.42d	306.21 ± 8.91c	485.49 ± 39.70b	455.22 ± 44.40b	772.66 ± 31.42a
Aldehydes										
Nonanal‐M	901.72 ± 35.72a	235.38 ± 6.27b	99.42 ± 8.88c	79.25 ± 1.18c	75.29 ± 7.40c	749.11 ± 13.39a	256.23 ± 14.35b	114.30 ± 6.70c	87.70 ± 7.88d	68.69 ± 7.37d
Nonanal‐D	413.59 ± 41.93a	130.30 ± 14.13b	78.61 ± 4.86c	79.17 ± 6.79c	85.60 ± 6.99c	277.11 ± 25.45a	129.45 ± 15.12b	91.67 ± 11.58c	84.51 ± 16.67c	79.35 ± 15.62c
Phenylacetaldehyde‐M	2,049.89 ± 18.72a	581.59 ± 14.96b	533.98 ± 21.69c	407.69 ± 28.34d	377.50 ± 10.83d	2,033.72 ± 20.30a	541.22 ± 42.13c	667.36 ± 20.72b	374.30 ± 3.80d	328.87 ± 25.21d
Phenylacetaldehyde‐D	1,640.78 ± 5.51a	253.47 ± 1.56b	217.62 ± 26.72c	160.80 ± 11.20d	137.49 ± 5.31d	1,559.10 ± 10.51a	244.21 ± 9.30c	302.99 ± 20.48b	182.33 ± 8.99d	116.76 ± 8.39e
E‐E‐2‐4‐Heptadienal	166.42 ± 8.89a	37.81 ± 2.19b	36.48 ± 1.42b	31.07 ± 5.03bc	24.95 ± 2.46c	234.56 ± 2.22a	29.63 ± 7.76c	42.18 ± 3.35b	33.16 ± 2.43c	24.95 ± 4.99c
E‐Z‐2‐4‐Heptadienal	207.79 ± 18.55a	153.22 ± 5.68b	159.16 ± 7.36b	156.15 ± 2.40b	113.49 ± 7.36c	286.46 ± 0.66a	142.60 ± 4.82d	192.44 ± 3.53b	170.30 ± 13.49c	138.35 ± 1.19d
Benzaldehyde‐M	228.13 ± 4.24a	114.96 ± 11.48b	99.94 ± 3.19c	95.88 ± 10.29c	101.64 ± 4.45bc	236.69 ± 11.75a	100.88 ± 9.11c	137.76 ± 8.25b	143.83 ± 3.41b	65.41 ± 2.06d
Benzaldehyde‐D	109.47 ± 8.82a	95.06 ± 4.54b	64.24 ± 5.37c	61.27 ± 2.75c	57.42 ± 2.08c	138.22 ± 3.63a	85.08 ± 2.85c	104.97 ± 6.17b	74.21 ± 5.06d	57.76 ± 3.35e
Methional‐M	372.66 ± 4.09a	90.70 ± 4.38b	50.42 ± 2.66c	40.37 ± 5.61d	37.83 ± 5.15d	374.08 ± 8.34a	82.70 ± 2.29b	86.40 ± 5.44b	60.24 ± 2.73c	30.72 ± 1.28d
Methional‐D	1,745.87 ± 5.70a	532.18 ± 8.10b	503.26 ± 36.28b	477.18 ± 26.30c	351.82 ± 32.74d	1,652.81 ± 11.12a	536.69 ± 4.06c	697.63 ± 21.16b	506.88 ± 2.91d	454.11 ± 6.13e
Furfurol	151.51 ± 4.35a	24.53 ± 1.35b	21.30 ± 2.53bc	20.33 ± 2.50bc	19.44 ± 6.30c	124.95 ± 7.69a	21.36 ± 4.67b	23.43 ± 2.09b	18.71 ± 1.83b	18.68 ± 4.29b
Hexanal‐M	157.50 ± 4.51a	20.09 ± 1.86b	19.06 ± 1.90b	17.76 ± 1.49b	16.75 ± 1.68b	139.62 ± 2.30a	19.68 ± 4.41b	22.23 ± 2.61b	20.10 ± 1.77b	17.51 ± 0.66b
Hexanal‐D	480.58 ± 36.06a	19.36 ± 2.35b	13.80 ± 1.57b	16.27 ± 1.63b	17.79 ± 3.39b	537.34 ± 63.31a	15.68 ± 0.013b	15.88 ± 3.34b	17.27 ± 2.20b	20.44 ± 4.49b
Butanal	385.21 ± 3.31a	72.40 ± 2.48b	72.51 ± 0.81b	58.45 ± 3.46c	65.88 ± 6.31b	350.60 ± 10.21a	70.63 ± 5.74b	63.33 ± 6.08b	33.20 ± 1.02c	62.34 ± 6.78b
Octanal	87.73 ± 6.93b	90.67 ± 1.82b	114.84 ± 13.25a	97.02 ± 11.21b	81.40 ± 2.17b	81.33 ± 6.15b	85.09 ± 4.12b	63.37 ± 2.66c	68.87 ± 9.45c	111.55 ± 7.24a
2‐Methylbutanal	395.26 ± 2.48b	761.38 ± 7.42a	362.78 ± 6.04c	359.56 ± 8.58c	355.68 ± 7.67c	399.05 ± 26.24d	800.30 ± 3.72a	569.86 ± 13.43b	481.04 ± 14.80c	185.03 ± 15.50d
3‐Methylbutanal	466.59 ± 1.29b	499.93 ± 4.13a	214.44 ± 6.35c	168.68 ± 13.57d	183.05 ± 9.27d	443.43 ± 8.87b	520.31 ± 29.67a	346.11 ± 18.29c	218.82 ± 18.43d	91.93 ± 4.49e
Alcohols										
1‐Hexanol	248.44 ± 3.18a	109.37 ± 7.07b	78.36 ± 7.53c	88.10 ± 2.52c	85.23 ± 5.52c	268.05 ± 5.30a	99.23 ± 8.09b	105.23 ± 3.03b	98.31 ± 1.83b	72.78 ± 4.52c
E‐2‐Hexenol‐M	259.58 ± 3.61a	78.81 ± 4.40b	64.97 ± 3.95c	53.88 ± 5.48d	80.76 ± 5.34b	281.19 ± 4.04a	63.70 ± 6.22c	81.93 ± 2.09b	52.92 ± 4.23d	48.09 ± 2.45d
E‐2‐Hexenol‐D	2,867.64 ± 36.09a	216.76 ± 2.47d	314.55 ± 10.02c	401.14 ± 12.39b	392.21 ± 12.87b	2,604.23 ± 27.02a	232.49 ± 7.89e	472.15 ± 28.12b	382.60 ± 13.48c	313.76 ± 7.33d
3‐Methylpentanol	217.96 ± 10.10a	8.72 ± 2.41b	7.78 ± 1.00b	8.30 ± 0.28b	9.15 ± 0.36b	239.95 ± 5.68a	6.57 ± 0.92c	12.59 ± 2.11b	8.38 ± 1.95bc	8.42 ± 0.98bc
1‐Pentanol	115.87 ± 3.14a	72.42 ± 3.47b	45.23 ± 0.77c	45.69 ± 1.92c	74.00 ± 5.20b	108.39 ± 4.93a	73.84 ± 5.51c	41.61 ± 3.03e	53.25 ± 7.89d	98.81 ± 2.81b
2‐Methylbutanol	726.89 ± 10.43a	71.20 ± 0.46c	94.15 ± 3.98b	54.59 ± 0.72d	68.58 ± 1.73c	769.92 ± 10.83a	61.32 ± 9.62c	71.37 ± 7.79c	61.53 ± 4.59c	120.63 ± 2.73b
3‐Methylbutanol	82.91 ± 2.44c	881.67 ± 75.86b	1,678.44 ± 33.45a	1,732.73 ± 60.22a	1,642.38 ± 51.01a	83.97 ± 4.37d	701.33 ± 8.50c	1,320.41 ± 121.77b	1,414.22 ± 122.67ab	1,478.50 ± 36.83a
1‐Butanol	2,057.89 ± 22.54a	159.23 ± 13.94c	213.62 ± 2.98b	215.31 ± 8.76b	204.30 ± 5.35b	2,003.53 ± 35.67a	163.69 ± 5.56c	256.78 ± 4.76b	183.54 ± 12.67c	157.86 ± 6.32c
2‐Methylpropanol	74.90 ± 1.80abc	83.12 ± 6.58a	64.10 ± 6.23c	71.23 ± 5.42bc	81.87 ± 7.78ab	67.38 ± 3.10b	80.79 ± 12.07a	78.07 ± 3.61a	79.10 ± 3.85a	54.66 ± 0.20c
Ethanol‐M	773.99 ± 13.26b	906.03 ± 11.51a	384.08 ± 13.28c	370.67 ± 19.75c	380.81 ± 2.68c	1,123.28 ± 51.23a	1,030.45 ± 104.34a	624.72 ± 33.53b	542.54 ± 16.16b	244.02 ± 16.41c
Ethanol‐D	88.59 ± 1.29d	544.47 ± 13.18c	1,885.69 ± 70.41a	1,831.05 ± 43.84a	1,573.56 ± 40.76c	136.04 ± 7.10d	667.01 ± 37.36c	1,457.70 ± 91.41b	1,389.34 ± 40.02b	1,779.19 ± 39.47a
Linalool	307.34 ± 11.22d	1,540.96 ± 30.63c	2,312.47 ± 38.35b	2,315.23 ± 21.20b	2,423. 01 ± 47.84a	339.16 ± 17.03c	1,549.49 ± 8.65b	2,416.06 ± 23.38a	2,490.05 ± 8.40a	2,503.09 ± 105.21a
1‐Octen‐3‐ol	88.72 ± 1.04b	126.64 ± 4.72a	69.07 ± 4.56c	58.37 ± 5.21d	71.75 ± 3.36c	79.98 ± 8.15c	132.44 ± 4.42a	107.83 ± 4.79b	85.97 ± 2.42c	49.27 ± 9.97d
Ketones										
Acetoin	1,068.65 ± 16.72d	1,247.25 ± 3.54c	1,471.69 ± 23.17b	1,556.51 ± 13.99a	1,458.45 ± 17.31b	1,052.56 ± 2.79d	1,229.49 ± 31.47c	1,539.82 ± 10.37a	1,540.08 ± 26.82a	1,405.61 ± 37.57b
Acetone	4,182.73 ± 43.16a	1,782.04 ± 28.14b	1,291.06 ± 19.61c	1,098.89 ± 39.74d	1,036.14 ± 68.25d	3,835.80 ± 34.11a	1,740.47 ± 24.98c	1,977.81 ± 136.24b	1,539.08 ± 28.29d	386.61 ± 4.58e
Cyclohexanone	301.37 ± 11.62d	425.41 ± 3.79a	377.72 ± 6.84b	289.24 ± 3.70e	318.22 ± 1.86c	306.27 ± 8.33a	327.62 ± 2.33b	380.17 ± 5.76a	281.61 ± 12.24c	209.76 ± 19.91d
6‐Methyl‐5‐hepten‐2‐one	237.37 ± 7.95a	112.55 ± 6.67b	66.75 ± 4.31c	66.60 ± 8.27c	67.71 ± 4.94c	275.64 ± 10.79a	89.15 ± 3.22c	100.01 ± 4.38b	82.24 ± 2.84c	53.16 ± 1.72d
Furans										
2‐Ethylfuran	151.09 ± 5.03d	174.04 ± 5.90d	231.90 ± 8.35c	443.32 ± 24.77b	484.44 ± 24.55a	232.79 ± 3.40d	160.65 ± 6.18e	431.02 ± 31.97a	351.12 ± 32.36b	299.73 ± 3.15c
2‐Pentylfuran	83.89 ± 6.23a	47.01 ± 6.85c	59.54 ± 1.53b	50.80 ± 5.36c	48.02 ± 3.19c	92.62 ± 9.18a	57.73 ± 6.06c	78.26 ± 3.88b	47.28 ± 4.18cd	42.22 ± 3.59d
2‐Acetylfuran	116.14 ± 2.52d	143.32 ± 7.09c	139.18 ± 2.80c	161.21 ± 10.90b	278.84 ± 14.15a	109.20 ± 6.03d	142.58 ± 4.92c	178.39 ± 3.22b	143.48 ± 6.29c	205.64 ± 20.76a
Acids										
2‐Methylpropionic acid	980.12 ± 30.62c	9,593.35 ± 149.27a	9,592.00 ± 30.64a	9,576.79 ± 72.2a	8,732.55 ± 186.26b	918.27 ± 39.00e	10,156.44 ± 82.03a	8,986.20 ± 110.58d	9,678.86 ± 74.85b	9,319.22 ± 82.58c
2‐Methylbutanoic acid	68.92 ± 2.18e	134.76 ± 3.51d	525.19 ± 12.09a	352.67 ± 2.62c	404.33 ± 8.15b	70.03 ± 6.08c	145.99 ± 18.00b	153.33 ± 22.79b	197.27 ± 28.50b	808.58 ± 53.04a
Pyrazine										
2‐6‐Dimethylpyrazine	249.48 ± 7.16a	100.61 ± 2.53b	84.91 ± 4.95c	63.36 ± 6.12d	68.36 ± 3.92d	237.88 ± 7.17a	93.85 ± 1.40c	115.23 ± 5.90b	61.01 ± 2.88e	77.49 ± 3.93d
Ether										
Dimethyl disulfide	1,684.55 ± 14.51a	286.21 ± 2.08b	58.01 ± 1.31d	66.06 ± 1.91d	90.91 ± 3.98c	1,623.68 ± 28.53a	271.58 ± 5.36b	105.17 ± 9.91c	88.40 ± 7.71c	98.90 ± 1.83c

Note

The significance analysis results were based on the peak volume value of FMP during NF and IF process, and different letters indicated significant differences (*p* < .05).

Abbreviations: D: dimer; IF: inoculated fermentation; IF‐0, IF‐6, IF‐12, IF‐18, IF‐24: IF samples were obtained on 0, 6, 12, 18, and 24 days; M: monomer; NF: natural fermentation; NF‐0, NF‐6, NF‐12, NF‐18, NF‐24: NF samples were obtained on 0, 6, 12, 18, and 24 days.

Esters, closely related to the fruity and sweet flavor, played an important role in FMP flavor. The change of eight esters during fermentation process is shown in Figure 5a and Table 2. Most of esters decreased with the increasing in fermentation time, including hexyl hexanoate, ethyl nonanoate, methyl octanoate, isoamyl hexanoate, and gamma‐butyrolactone‐M. High‐acid environment might promote the hydrolysis of above esters (Tomita et al., 2018). In the early fermentation, isoamyl hexanoate‐M sharply deceased, but the isoamyl hexanoate‐D sharply increased. This phenomenon indicated that high content of isoamyl hexanoate‐M turned into the isoamyl hexanoate‐D, which was in accordance with the result of fingerprint analysis. In addition, isoamyl hexanoate posed an acid stability and was not easy to hydrolyze at low pH. Hence, isoamyl hexanoate content was the highest in all the eaters during fermentation process. Wang, Wang, Xiao, Liu, Deng, et al. (2019) found that ethyl salicylate, ethyl palmitate, methyl linoleate, ethyl linoleate, and ethyl stearate were the main esters in FMP during fermentation process, but no many isoamyl hexanoate was produced. The reason for the difference in two experiments was in accordance with fermentation conditions, salt content, and pepper varieties. According to these results, it was speculated that isoamyl hexanoate played an important role in the aroma quality of FMP. Ethyl acetate, with pineapple flavor, showed an increased tendency during fermentation process. Microorganisms turned the fermented carbohydrates into the end products during fermentation process, including organic acids and alcohols (Kim et al., 2016). The organic acids and alcohols could combine each other to form several esters, such as ethyl acetate (Wang, Wang, Xiao, Liu, Deng, et al., 2019). It was found that more ethyl acetate was produced during liquor fermentation, and became a key volatile compound of liquor during liquor brewing process (Cai et al., 2019). However, little research reported that ethyl acetate could increase to high level during vegetable fermentation. Hence, ethyl acetate and isoamyl hexanoate were the two main esters at the end of fermentation, and attributed to the FMP flavor.

Aldehydes showed lower threshold values and strong fragrance‐giving ability. For FMP, aldehydes with suitable content were beneficial for the maintenance of pungency flavor. Eight kinds of aldehydes were selected to explore the change in aldehydes during fermentation process, as shown in Figure 5b and Table 2. Most aldehydes decreased with the prolonging of fermentation time, including methional, phenylacetaldehyde, and furfurol, especially for methional and phenylacetaldehyde. During fermentation process, the aldehydes turned into the acids and alcohols under the action of microorganisms, which was an important reason for aldehyde degradation. Meanwhile, condensation could be occurred among aldehydes (Sukharev, Mariychuk, Onysko, Sukhareva, & Delegan‐Kokaiko, 2019), which accelerated aldehyde degradation. The decrease in above aldehydes weakened the pungency of FMP at some extent. Zhao et al. (2015) found that almost all aldehydes, such as butanal, octanal, and others, decreased with the prolonging of pumpkin juice fermentation time. For 2‐methylbutanal, the content increased in the first 6 days, then decreased in the late 18 days. Li, Dong, et al. (2016) explored the relationship between bacterial communities and volatile compounds in red pepper paste, and found that *Pseudomonas* posed an obvious correlation with 2‐methylbutanal. Hence, it was speculated that the change in 2‐methylbutanal content might be related to *Pseudomonas* during fermentation process. Hazelwood, Daran, van Maris, Pronk, and Dickinson (2008) found that some aldehydes, such as methylbutanal, could be generated by Strecker degradation of methionine and leucine or the Ehrlich pathway. Although octanal content slightly increased during fermentation process, the content was still lower than most other aldehydes. At the end of fermentation, the content of all aldehydes was low, which weakened the pungency of FMP.

Alcohols, with high threshold values, were mainly generated by alcohol and lactic acid fermentation (Wang, Wang, Xiao, Liu, Deng, et al., 2019). However, the threshold of unsaturated aldehyde was low, which made many contributions to food flavor (Lorenzo, Carballo, & Franco, 2013). As shown in Figure 5c and Table 2, the content of some alcohols increased sharply in the early fermentation time, and then maintained a stable level in the late fermentation time, including linalool, ethanol‐D, and 3‐methylbutanol. It indicated that the ability in early fermentation was stronger than that in late fermentation. In early fermentation time, LAB became the prominent microorganism quickly comparing with other microorganisms, promoted LAB fermentation, and produced some acids and alcohols. Nguyen et al. (2013) also proved that LAB in traditional fermented vegetables of Vietnam could grow rapidly and became the dominant bacteria under suitable conditions during fermentation process. In addition, alcohol fermentation also existed in early fermentation process, and produced some alcohols, especially for ethanol. In late fermentation time, the fermentation ability of microorganism was weakened because of the worst living conditions, such as less nutrient substance (Beganovi et al., 2014). However, some alcohols showed obvious decrease in tendency in early fermentation time, then hardly changed, especially for E‐2‐hexenol‐D and 1‐butanol. E‐2‐hexenol‐D was sensitive to the acids, and easily decomposed or turned into other compounds at low pH. The reason for 1‐butanol degradation might be that 1‐butanol turned into butyric acid at the action of microbial metabolism and oxidation. The increase in 2‐methylbutanoic acid was probably related to above change. At the end of fermentation, linalool content was the highest in all alcohols, and gave flower‐like aroma, which posed an important contribution to the FMP flavor. Linalool was also existed in some fermented vegetable products, such as fermented red pepper pastes and cucumber (Li, Dong, Zhao, & Zhu, 2020; Zhou & McFeeters, 1998). Zhou and McFeeters (1998) found that the content of linalool in fermented cucumber increased to several times than its odor threshold during fermentation.

The other volatile compounds, including 4 ketones, 3 furans, 2 acids, 1 pyrazine, and 1 ether, are shown in Figure 5d,e and Table 2. The content of 2‐methylpropionic acid and 2‐methylbutanoic acid increased after fermentation. In particular for 2‐methylpropionic acid, the content sharply increased to a high level. Whatever NF or IF, lactic acid fermentation was the main fermentation pathway, and involved the oxidation of carbohydrates to other compounds, which caused the production of lots of acids. Li et al. (2020) explored the effect of different salt content (10%, 15%, 20%, and 25%) on microbial categories and related qualities, and found that LAB became the prominent population at high salt content. At the end of fermentation, it was found that the pH value was up to about 4.10, which was similar to our experiment. Ketones possessed a lower threshold value and more contributed to food flavor. Acetone, cyclohexanone, and 6‐methyl‐5‐hepten‐2‐one decreased with the prolonging of fermentation time. In particular for acetone, the peak value decreased from 4,009.27 to 711.38 in the whole fermentation. Sukharev et al. (2019) found that ketone could condensate with aldehydes or other ketones, and condensation reaction might be the main reason for the decrease in ketones during fermentation process. The final peak values of 2‐ethylfuran, 2‐pentyfuran, and 2‐acetylfuran were about 2 times than those of the initial ones. It indicated that some furans were generated during fermentation process. Furans were hardly detected in many fermented foods (Kim et al., 2019). It is indicated that 2‐ethylfuran, 2‐pentyfuran, and 2‐acetylfuran were probably the characteristic volatile compounds in FMP. Only 1 pyrazine and ether were detected. Hence, it was difficult to analyze the change in pyrazine and ether during fermentation process.

As shown in Figure 5a_1_–d_1_, it was found that there was similar content in volatile compounds between NF and IF samples. Under suitable fermentation conditions, including temperature, pH, O_2_, the LAB fermentation was the prominent pathway whatever NF or IF (Sanlier et al., 2017). Many other microorganisms were inhibited by LAB. Hence, two fermentation methods produced the similar volatile compounds, but IF might shorten fermentation period and promote FMP quality. It was proved that the use of inoculated microorganism starters guaranteed the agreeable sensory properties, including volatile compounds (Woutets et al., 2013; Zhao et al., 2015).

In summary, main volatile compounds, including esters, aldehydes, alcohols, and acids, notably changed during fermentation. Almost all esters, aldehydes, and some alcohols posed an obvious decrease, and some alcohols and all acids posed an obvious increase during fermentation process. These results were related to microbial fermentation. However, there were no obvious differences in volatile compounds between NF and IF samples. Hence, further analysis was needed to explore volatile compounds during NF and IF process.

### 3.5 Analysis based on PCA results

**FIGURE 6:**
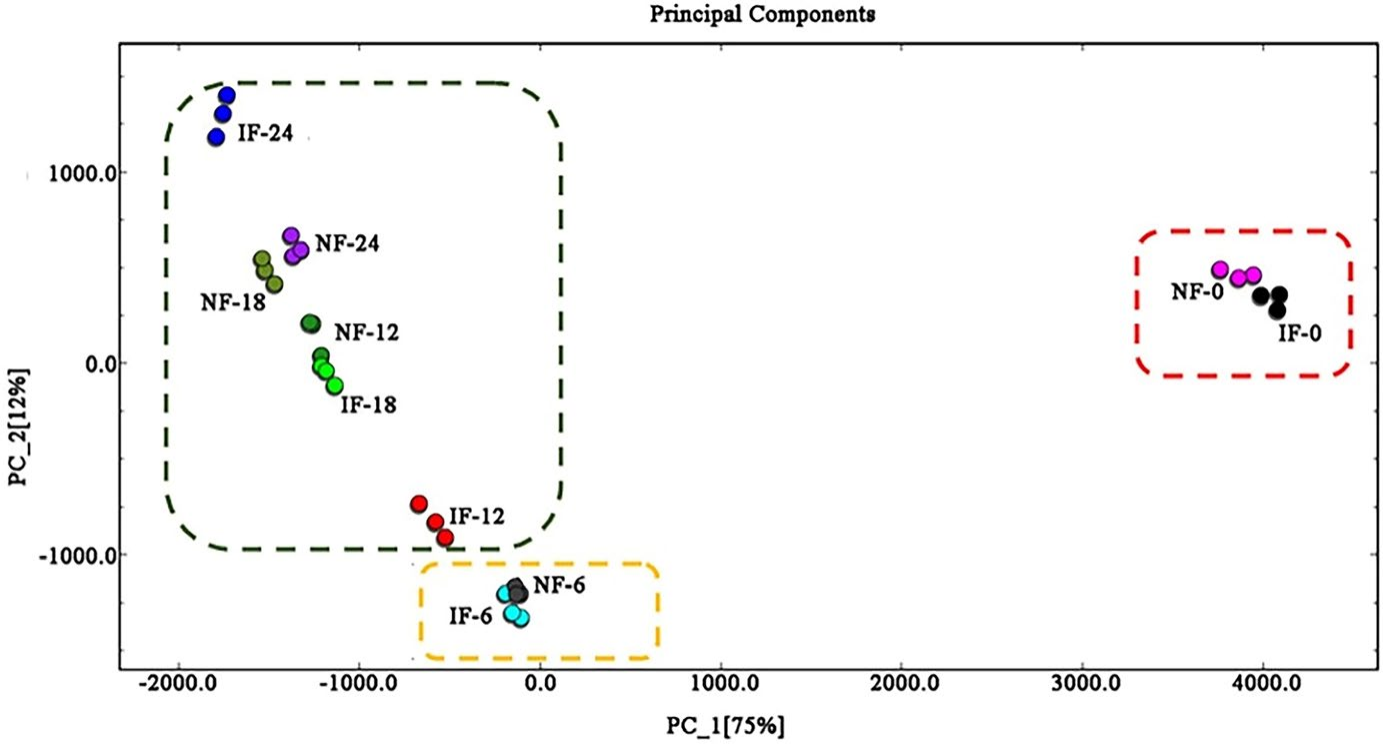
PCA of volatile compounds during NF and IF process. IF: inoculated fermentation; IF‐0, IF‐6, IF‐12, IF‐18, IF‐24: IF samples were obtained on 0, 6, 12, 18, and 24 days; NF: natural fermentation; NF‐0, NF‐6, NF‐12, NF‐18, NF‐24: NF samples were obtained on 0, 6, 12, 18, and 24 days

Principal component analysis, a multivariate statistical analysis, was used to turn original correlated variables into linearly uncorrelated variables by several related transformation. These uncorrelated variables obtained by PCA could reflect the relationships among the observed variables (Cirlini et al., 2019; Yao et al., 2015). The correlated variables could be distinguished by the positive or negative score values in PC1 and PC2. Yilmaztekin and Sislioglu (2015) found that most important volatile compounds in fermented and raw European cranberrybush were divided into four uncorrelated parts by PCA, and PCA discriminated the fermentation stage as three groups. In our research, PCA obtained by original date was used to compare the difference in principal compounds (Figure 6). An obvious separation of two principal compounds was found during fermentation process and two fermentation methods (NF and IF). Two principal compounds (PC1 and PC2) were up to 87% in variation of originate date. PC1 was up to 74%, but PC2 was only up to 13% in variation of originate date. As shown in Figure 6, the samples showed a well separation degree each other, and three parts were presented in PCA based on previous analysis and heat map. The higher the separation degree, the lower the correlation in different samples. Samples in different parts posed low correlation. According to PCA, an obvious difference was found comparing samples on 0 days (NF and IF) with other samples (NF and IF), as shown in red frame. PC1 and PC2 of samples on 0 days were the positive score values. The result indicated that fermentation time played a notable influence on volatile compounds of FMP. The other samples could be well distinguished based on the score values of PC1 and PC2. The samples on 6 days were clustered in the yellow frame, but few differences were found between NF and IF samples. Principal compounds of samples on 12, 18, and 24 days of NF and IF were clustered in green frame. The samples on 12 and 18 days during IF have negative score values of PC1 and PC2, but the negative score values of PC1 and the positive score values of PC2 in other samples that were found. According to above results, it was found that there were few differences in principal compounds in the late fermentation time. It is indicated that the late fermentation ability was weakened because of less nutrition substance. In summary, the PCA well discriminated the stage of NF and IF as three groups (0 days; 6 day; and 12, 18, and 24 days). Based on above results, fermentation time played a key role in change of volatile compounds, but the two fermentation methods posed little effect on volatile compounds.

### 3.6 Clustering analysis based on heat map

**FIGURE 7:**
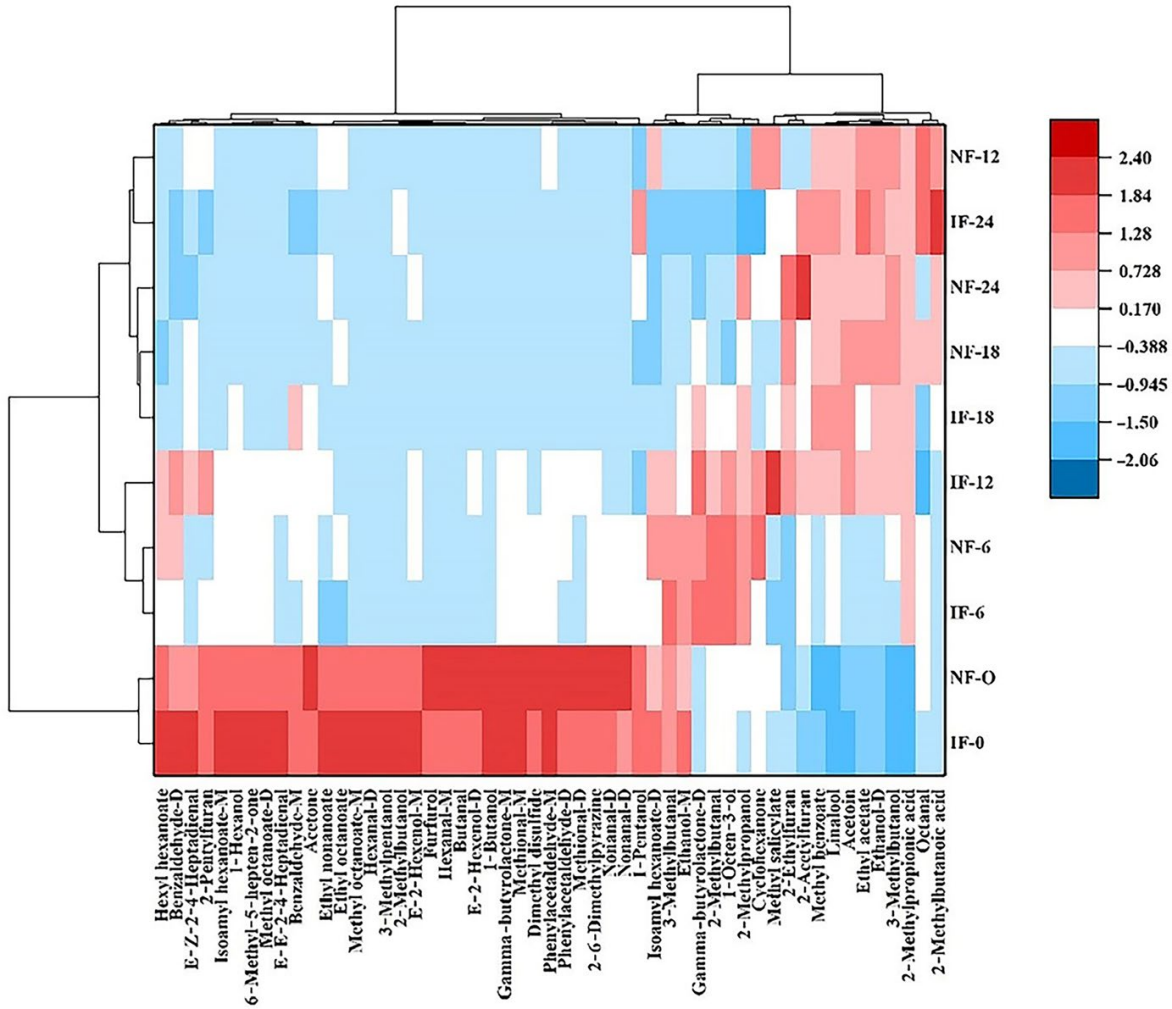
Heat map clustering of volatile compounds during NF and IF process. D: dimer; IF: inoculated fermentation; IF‐0, IF‐6, IF‐12, IF‐18, IF‐24: IF samples were obtained on 0, 6, 12, 18, and 24 days; M: monomer; NF: natural fermentation; NF‐0, NF‐6, NF‐12, NF‐18, NF‐24: NF samples were obtained on 0, 6, 12, 18, and 24 days

To further analyze the differences in volatile compounds of FMP during fermentation process, the cluster analysis was obtained based on the heat map, as shown in Figure 7. In the heat map, the samples were clustered in the horizon, and volatile compounds were clustered in the vertical. There was the high correlation degree in the same category. The samples showed a higher correlation degree each other with the shortening of Euclidean distance. Based on the vertical mode, all samples were clustered into three main categories. Samples on 0 days were clustered together under NF and IF, as the first category. Hence, NF samples were highly similar with IF ones on 0 days. According to the color depth, samples on 0 days showed high content of volatile compounds, such as hexyl hexanoate, methyl octanoate, gamma‐butyrolactone, phenylacetaldehyde, dimethyl disulfide, and acetone. The second category was the NF and IF samples on 6 day. The other samples presented a high correlation between NF (12–24 days) and IF (12–24 days), as the third category. In the late fermentation time, several volatile compound content was up to a high level, including 2‐methylbutanoic acid, 2‐methylpropionic acid, 2‐acetylfuran, and ethyl acetate. On the basis of vertical analysis in heat map, it could be concluded that volatile compounds changed with the prolonging of fermentation time, especially in the early fermentation time. Above results were similar with PCA and previous analysis. Based on the horizontal mode, all volatile compounds in FMP could be classified into two main categories according to the variation tendency (increase and decrease). In heat map, the content of hexyl hexanoate, methyl octanoate, gamma‐butyrolactone, E‐2‐hexenol, 2‐methylbutanol, dimethyl disulfide, acetone, and 1‐butanol obviously decreased to a low level. Apparently, these volatile compounds were clustered into a category with the shortening of Euclidean distance, as the first category. The result was related to change in microorganisms during fermentation process. However, methyl benzoate, ethyl acetate, linalool, 3‐methylbutanol, acetoin, 2‐ethylfuran, 2‐acetylfuran, 2‐methylpropionic acid, and 2‐methylbutanoic acid were clustered into another category, showing an increase tendency in content. LAB fermentation could turn the carbohydrate to other end products, such as acids and alcohols, so promoted the increase in above volatile compounds. These results from heat map further confirmed that HS‐GC‐IMS was a reliable way to detect the volatile compounds in FMP during NF and IF periods.

## 4 CONCLUSION

The study investigated the change in volatile compounds of FMP during NF and IF process using the HS‐GC‐IMS instrument and related supplementary analysis software. A total of 53 volatile compounds were identified in all samples, including 12 esters, 17 aldehydes, 13 alcohols, four ketones, three furans, two acids, one pyrazine, and one ether. With the prolonging of fermentation time, most esters, aldehydes, and alcohols obviously decreased, especially for isoamyl hexanoate, methyl octanoate, gamma‐butyrolactone, phenylacetaldehyde, methional, and E‐2‐hexenol. However, 2‐methylbutanoic acid, 2‐methylpropionic acid, linalool, and ethyl acetate increased to a high level during fermentation process. Above volatile compounds were the indicators of flavor at the end of fermentation time, playing a key role in unique flavor of FMP. Hence, the fermentation time possessed an obvious effect on change in volatile compounds. However, volatile compounds in NF and IF samples showed slight differences at the same fermentation time. Based on PCA and heat map, the fermentation process in all samples was well discriminated as three stages (0 days; 6 day; and 12, 18, and 24 days), and all volatile compounds were divided into two categories (increase and decrease). Above results proved that HS‐GC‐IMS was an effective method to analyze the change in volatile compounds in FMP during NF and IF process.

## CONFLICT OF INTEREST

The authors declare that they do not have any conflict of interest.

## HUMAN AND ANIMAL RIGHTS

No animals or humans were used for studies that are the basis of this manuscript.
